# Predicting melanoma survival and metastasis with interpretable histopathological features and machine learning models

**DOI:** 10.3389/fmed.2022.1029227

**Published:** 2023-01-06

**Authors:** Justin Couetil, Ziyu Liu, Kun Huang, Jie Zhang, Ahmed K. Alomari

**Affiliations:** ^1^Department of Medical and Molecular Genetics, Indiana University School of Medicine, Indianapolis, IN, United States; ^2^Department of Statistics, Purdue University, West Lafayette, IN, United States; ^3^Department of Biostatistics and Health Data Science, Indiana University School of Medicine, Indianapolis, IN, United States; ^4^Department of Pathology, Indiana University School of Medicine, Indianapolis, IN, United States

**Keywords:** computational pathology, histopathology, biomedical image processing, melanoma, neoplasm metastasis, survival prognosis, metastatic prognosis

## Abstract

**Introduction:**

Melanoma is the fifth most common cancer in US, and the incidence is increasing 1.4% annually. The overall survival rate for early-stage disease is 99.4%. However, melanoma can recur years later (in the same region of the body or as distant metastasis), and results in a dramatically lower survival rate. Currently there is no reliable method to predict tumor recurrence and metastasis on early primary tumor histological images.

**Methods:**

To identify rapid, accurate, and cost-effective predictors of metastasis and survival, in this work, we applied various interpretable machine learning approaches to analyze melanoma histopathological H&E images. The result is a set of image features that can help clinicians identify high-risk-of-metastasis patients for increased clinical follow-up and precision treatment. We use simple models (i.e., logarithmic classification and KNN) and “human-interpretable” measures of cell morphology and tissue architecture (e.g., cell size, staining intensity, and cell density) to predict the melanoma survival on public and local Stage I–III cohorts as well as the metastasis risk on a local cohort.

**Results:**

We use penalized survival regression to limit features available to downstream classifiers and investigate the utility of convolutional neural networks in isolating tumor regions to focus morphology extraction on only the tumor region. This approach allows us to predict survival and metastasis with a maximum F1 score of 0.72 and 0.73, respectively, and to visualize several high-risk cell morphologies.

**Discussion:**

This lays the foundation for future work, which will focus on using our interpretable pipeline to predict metastasis in Stage I & II melanoma.

## 1. Introduction

Melanoma is the fifth most common cancer, with about 110,000 new cases in the US alone in 2021, and its incidence is increasing approximately 1.4% each year. Most melanoma patients are considered cured when their superficial, thin, primary melanoma (Stage I) is surgically removed, resulting in a 99.4% 5-years survival rate (1). However, melanoma can recur as a locoregional disease or distant metastases in 6% of Stage I patients where cancer is limited to the superficial dermis, and in 20% of Stage II patients where cancer has invaded the deeper dermis and subcutis (2). Currently, clinicians still do not have accurate and cost-effective ways to predict tumor recurrence and metastasis from the primary tumor histopathological images of early-stage patients. In addition, current melanoma staging systems depend primarily on histopathologic features, and sometimes involve invasive sentinel lymph node biopsies. These procedures have not been shown to improve prognosis for early-stage patients (tumor invasion < 1 mm) and therefore expose patients to unnecessary morbidity (3).

Currently, prognostication of localized melanoma (i.e., no distant metastases) relies on several histopathological criteria established by pathologists’ examination of hematoxylin and eosin (H&E) stained tissue sections. These include Breslow depth and the presence of ulceration and microsatellitosis. Moreover, it also depends on the identification of tumor deposits in sentinel lymph nodes in cases where such procedure is performed. Tumor ulceration is the loss of full-thickness epithelium above the growing tumor and is an independent prognostic factor. Integrating these histopathologic findings with clinical information like the site of origin for tumors is important: acral (non-sun-exposed regions) and lentigo maligna melanomas both could have fusiform cells, but the prognoses are different, with thickness-matched acral melanoma being more aggressive (4). Moreover, prognosis varies by histologic subtype, where nodular and acral have generally worse outcomes than thickness-matched superficial spreading and desmoplastic subtypes (5, 6). However, personalized prognostication of early-stage melanoma (< 0.75 mm) remains suboptimal. Ulceration, the hallmark of poor prognostic feature, is not a common finding for early-stage melanoma, and though most literature suggests that lymphocyte infiltration is an important marker for better prognoses, this relationship is uncertain for lesions under 0.75 mm depth of invasion (7).

Because histopathologic features remain suboptimal in predicting melanoma prognosis in early-stage patients, and early-stage patients make up about 80% of all newly diagnosed cases of melanoma (8), there is an pressing need for developing a machine learning based computational pathology pipeline to stratify patients. Rigorous measurement of cellular/nuclear morphological features of primary tumor pathological images may provide consistent performance across the heterogenous landscape of melanoma. Currently, published machine learning models using H&E images to study melanoma prognosis are mostly “black-box” models based on deep neural networks, specifically Convolutional Neural Networks (CNNs) (9). For instance, Forchhammer et al. applied CNNs trained on whole slide images to establish a model that stratified patients by their 10-years survival rates; however, improving risk classification beyond the existing staging guidelines has proven difficult for early-stage patients (10). CNN-based approaches have also been used to predict survival using locoregional/metastatic biopsies (11), which applies to less than 20% of all melanoma patients (12). Furthermore, these deep learning-based models identified abstract features that are neither visible nor directly associated with human-interpretable cell morphology and tissue structure, which is a major barrier for clinical adoption and generation of new hypotheses for research. It is imperative that pathologists and researchers understand the mechanisms behind the disease progression. In this paper, we present a pipeline with more interpretable machine learning methods that can be used alongside the very accurate, but less interpretable deep learning techniques.

Kulkarni and Robinson (13) published the only histopathology-based melanoma metastasis model to date. They achieved impressive accuracy (88–90%) for high/low risk stratification based on their deep learning models. Due to the lack of interpretability of the neural network, ablation studies were adopted to show that the ratio of lymphocyte area over tumor cell area was crucial for model accuracy. The individual contributions of the rest of the morphology feature set were not readily apparent. When it is difficult to understand what information the neural networks rely on to make their prediction, it is more difficult for pathologists, clinicians, and researchers to investigate further. This is a bottleneck for effective translational application of these neural networks. In addition, this work provided very accurate classification for patients with more advanced disease (skewed toward Stages II–III). However, Stage I patients comprise the majority of the general melanoma population, and metastasis is most likely to be missed in these individuals.

The work we present herein focuses on developing a machine learning pipeline to identify the reliable and interpretable H&E histopathology image features to predict 5-years survival and metastasis using the primary site biopsies from Stages I, II, and III melanoma patients. We have demonstrated that simple machine learning models (i.e., logistic regression, k-Nearest Neighbors, support vector machines, and random forest classifiers) using extracted interpretable features of cellular and nuclear morphology can generated accurate, sensitive, and specific prediction for 5-years survival and metastasis risks. We first applied deep learning methods (14) to identify tumor regions with CNN models, and extracted interpretable morphological features from only the tumor regions, and understand how this impacts downstream classifier performance. Because some of the morphological descriptors we used can be correlated to each other, we applied LASSO Cox regression to reduce the number of image features available to downstream classifiers to reduce the likelihood of overfitting and improve ease of interpretation by a pathologist.

One of the challenges for our study is that samples from large cancer databases, such as The Cancer Genome Atlas (TCGA), Clinical Proteomic Tumor Analysis Consortium (CPTAC), provide almost only survival information, without any metastasis information for Stages I and II patients. To tackle this challenge, we approached our work in two steps: (1) Training machine learning models for survival prediction with a merged cohort form the TCGA, CPTAC, and our own curated, high-quality local IU School of Medicine (IUSM) cohort; and (2) further refining it to predict melanoma metastasis on the IUSM cohort.

We demonstrated that our identified H&E image features can serve as accurate, rapid, and low-cost predictors of metastasis. Further, this approach can be seamlessly integrated into clinical workflows, given that digitized biopsies are an approved diagnostic tool (15) and interpretation of biopsies by a pathologist is standard of care in melanoma. To summarize, our work begins to bridge a significant clinical and research gap: the need for an adoptable and interpretable cell morphology machine learning pipeline to work alongside deep-learning approaches in the study of melanoma metastasis.

## 2. Data and materials and methods

### 2.1. Data description

To maximize sample size and test the model generalizability, we applied our pipeline to three melanoma cohorts: The Cancer Genome Atlas (TCGA) cohort, the Clinical Proteomic Tumor Analysis Consortium (CPTAC) cohort (16), and the Indiana University School of Medicine (IUSM) cohort. For all three, we restrict our analyses to Stages I, II, and III melanoma patients. Stage IV patients, who already have distant metastases, were excluded. Further, slides that are misdiagnoses, microsatellite metastases, and those that cannot be confirmed as primary site biopsies due to lack of visible intact epidermis were removed. This rigorous quality control resulted in a sample size of 81 whole slide images (WSI) from 71 patients in the TCGA cohort, and 45 WSI from 19 patients in the CPTAC cohort. The TCGA and CPTAC cohorts only contain survival information, with no metastasis information. The IUSM cohort had 92 WSIs from 70 patients with both metastasis and survival information. This information is summarized in Table 1.

**TABLE 1:** Clinical follow up and slide quality among Stages I–III patients in three patient cohorts.

	Cohort; # patients (# slides)			
	Survival			Metastasis
	TCGA	CPTAC	IUSM	IUSM
Stages I, II, and III patients	129 (145)	63 (117)	70 (92)	70 (92)
Adequate quality slides	71 (81)	19 (45)	70 (92)	70 (92)
Follow up information (patients)				
Low risk–no event before 5 years	3	0	43	30
High risk–event before 5 years	7	8	7	26
Non-informative censoring before 5 years	61	11	20	14

### 2.2. Feature extraction pipeline

**FIGURE 1:**
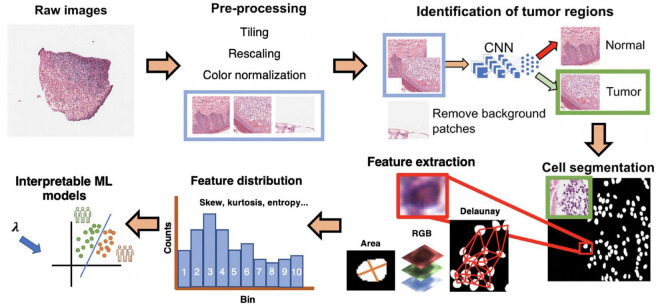
Interpretable cell morphology and machine learning pipeline. Whole slide images are tiled into 512 × 512 pixel patches and then rescaled for differences in resolution between datasets, background patches are removed by manually engineered color features, and tumor/normal regions are identified using a convolutional neural network that was trained with pathologist annotations. Tiles then normalized for hematoxylin and eosin (H&E) staining, and cells segmented with StarDist. Cell masks are analyzed with MATLAB to generate measures of cell morphology (e.g., area), tissue architecture (i.e., Delaunay distance), and identify small, hyperchromatic cells. 50 processed slides are randomly pooled, and k-means clustering (*k* = 10) is used to bin the distribution of each image feature and five distribution statistics are calculated (e.g., skew, kurtosis, mean). Finally, every cell of every slide is assigned the “bins” to generate a final dataset of N patients × 135 features.

Predicting metastasis and survival are two distinct but related tasks. Herein, we use the same feature extraction pipeline to predict 5-years metastasis for IUSM patients, and 5-years survival in the IUSM, TCGA, and CPTAC datasets (Figure 1). We modified the morphological feature set described in (17) by focusing on the morphological features and introducing two additional features (quantity and density) to describe lymphocytes and other small, hyperchromatic cells (e.g., pyknotic nuclei), each with 10 bins and five distribution statistics. We call this category *Small-Hyperchromatic cells*. In total, we have 135 morphological features extracted from WSIs to quantify the cell size and shape, as well as *Small-Hyperchromatic cell* density and counts, as well as statistics describing the distribution for each of these image features within each WSI (i.e., mean, standard deviation, skewness, kurtosis, and entropy).

#### 2.2.1. H&E whole slide image pre-processing

Before image normalization and feature extraction, we first rescaled WSIs from different cohorts to the same resolution. The TCGA and the CPTAC images are scanned using different resolutions, so all image patches were resized to match the IUSM cohort resolution of 0.25 microns-per-pixel (mpp), which corresponds to a 400x magnification. For the TCGA and CPTAC cohorts, we tiled images into squares with dimension of 512px * 0.25/mpp, and then rescaled them into 512 × 512 pixels. To filter out black and white background patches from the WSI, we removed patches where the mean intensity of RGB channels together was greater than or equal to 230, less or equal to 40, or with a standard deviation less than 20. This removed both black and white background patches. IUSM images required further processing, using the following criteria to remove black and white patches: red channel mean intensity being 90% or less than the blue channel mean intensity, red channel mean intensity being less than 170, and green channel mean intensity being greater than 210. The results of these criteria were visually inspected for accuracy and consistency among the three datasets. We then applied the color normalization algorithm proposed by Macenko, Neithammer (18) to avoid batch-effects both within and across datasets. This algorithm is unsupervised and based on singular value decomposition of opacity density values. Cells in the WSI from all three cohorts are segmented using StarDist, which uses all three RGB color channels to segment cells (19). StarDist was better suited to this task than hierarchical multilevel thresholding based on our evaluation (Supplementary Figure 1). We further removed background patches by filtering out those that had fewer than certain number of cells segmented by StarDist. For the CPTAC and IUSM slides, the cutoff is 15 cells, and for the TCGA, the cutoff is 10. The results of this preprocessing were visually verified by our pathologist.

#### 2.2.2. Identification of tumor regions with convolutional neural networks

**FIGURE 2:**
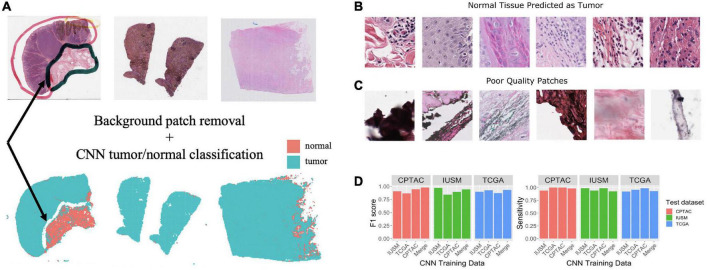
Training and assessment of convolutional neural network. Workflow and assessment of convolutional neural network. **(A)** Whole slide image patches are first filtered to remove background patches. This step removes artifacts, demonstrated by the ink from the whole slide images (WSI) that is not retained in the final set of patches (arrow). **(B)** Patches confused by the convolutional neural networks (CNN) can show mitotic figures and dense lymphocytic infiltrate. **(C)** Despite our filtering steps, there remain some artifact-ridden poor-quality patches. **(D)** Performance at epoch 200 for CNNs trained on the IU School of Medicine (IUSM), The Cancer Genome Atlas (TCGA), Clinical Proteomic Tumor Analysis Consortium (CPTAC), and merged datasets, comparing the Fl scores and sensitivity for tumor/normal classification achieved on validation sets from the IUSM, TCGA, and CPTAC cohorts.

Tumor biopsies contain variable amounts of normal tissue, therefore using the entire WSI to predict clinical outcomes may introduce additional bias. To study this, we focused our analysis on regions of the bulk tumor by using a CNN to triage tumor vs. normal image patches, and then applied our interpretable feature extraction pipeline to the CNN-identified tumor patches. The quality of specimen from the TCGA, CTPAC, and IUSM cohorts were very different. The IUSM cohort had the best quality, followed by TCGA and then CPTAC. We trained three different CNN’s–one for each cohort–to understand how varying data quality impacted tumor vs. normal image patch classification on the entire (Figure 2A), we describe training process and model architecture.

To train and validate these CNNs, our pathologist used QuPath (20) to manually annotate normal tissue and tumor regions on WSIs from all three cohorts. Respectively, 20, 19, and 90 WSIs were annotated for the TCGA, CPTAC, and IUSM cohorts. We cropped image tiles from all three cohorts, resized if necessary to match resolutions (described previously), and assigned tumor/normal labels to image patches using our pathologist’s QuPath annotations of tissue regions. Finally, we performed five-fold cross-validation using 1,000 tumor and 1,000 normal image tiles for training and validation (80/20% split). To ensure fair assessment of accuracy, we designed random sampling such that validation image tiles were not pulled from patients that appeared in the training set. It is important to note that some patients had multiple WSIs; therefore, naïve random sampling would mean that WSIs from a single patient could end up in both the testing and validation datasets. To prevent this, we ensured that the testing and validations splits were based on patients, not WSIs.

One of the most widely used CNN architectures for image classification and object detection is the “Inception” module, which employs convolutional kernels with different sizes, called scale filters (21). With our simple task and ample training data, we adopted the naïve version of “Inception” based model, herein called GoogLeNet, and modified the final layer of the network for binary tumor/normal tissue classification. We applied the ADAM (22) optimizer with learning rate 0.0002. Four different GoogLeNet models were trained using the CPTAC, IUSM, TCGA cohorts, and a final “Merged” cohort with 1,000 labeled patches from all three cohorts. Experimental results demonstrated that the GoogLeNet models achieve reasonable accuracy on all four validation datasets (> 0.8).

#### 2.2.3. Cell-level feature extraction, aggregation, and investigation

We have previously developed a morphological feature extraction pipeline for H&E images, as described by Cheng, Zhang (17), and adopt it here to predict melanoma outcomes. For each WSI, we first used the *regionprops* function in MATLAB version R2022a to calculate area, major and minor axis length of the cells, major/minor axis ratio, staining intensities (RGB three color channels), and described the density of cells in the image by measuring the minimum, maximum, and mean distance to neighboring cells. Staining intensities were not use as image features, but were used to engineering a new cell category, *Small-Hyperchromatic cells*. This process is described later. The neighbor relationship was defined using the Delaunay triangulation method among cell centroids.

Once all WSIs were processed, we randomly sampled 50 images from the three datasets (IUSM, CPTAC, and TCGA), to perform k-mean clustering with 10 clusters for every image feature. This is analogous to generating 10 *“bins”* for a histogram. These 10 bins for each category of features represent a dataset-wide census of cell morphology and maintain representations of heterogeneity that simple statistics such as average cannot. Five statistics on the distribution of these histograms were calculated: mean, standard deviation, skewness, kurtosis, and entropy. This gave us a summarized data structure of 7 features × (10 bins + 5 statistics) = 105 cell features per patient. If a patient had multiple WSIs, we calculated the mean of all feature values across the WSIs, providing a single patient-level vector.

In addition to these 105 features, we engineered cutoffs to identify a specific category of small cells with hyperchromatic staining, which we refer to as *Small-Hyperchromatic cells*. This category tends to represent necrosis and dense inflammation by highlighting lymphocytes, and pyknotic nuclei. *Small-Hyperchromatic cells* were defined by an area less than 450 pixels and a ratio of the long and short cell axes less than 2 (favoring round cells rather than spindle cells). We calculated the quantity and density (proportion of these cells to all cells in each image patch) of these small-hyperchromatic cells for an additional 30 features: 10 bins and 5 statistics for the quantity and density of small hyperchromatic cells each. Adding this to the previously described 105 features provided a total of 135 features per WSI.

### 2.3. Univariate feature analysis

To investigate the ability of individual image features to stratify patients, we took the approach described by Lu, Xu (23), iterating through 100 cutoffs in the range of values for each image feature, which generates two strata for which to calculate Kaplan-Meier estimates and extract FDR-adjusted *p*-values. The resulting significant cutoffs are used to generate Kaplan-Meier curves for survival and metastasis. This analysis conducted in R v4.1.0 using packages survival v3.2-13 (24) and survminer v.0.4.9 (25).

### 2.4. Multivariate supervised classification for risk stratification

Despite merging data from three different patient cohorts, our sample size is still limited due to stringent quality control and a focus on Stages I through III. In the setting of a high feature dimension and small sample size, we use Lasso Cox regression from the glmnet v4.1-1 package in R (26) to reduce collinearity in the downstream supervised classification task (Supplementary Figure 2). The trained Lasso Cox model provides two feature sets based on the accuracy metric of concordance: “1se” feature set for a model whose variance is heavily regularized, where cross-validation accuracy is within one standard error of the maximum accuracy; and the “min” feature set corresponds to the model with the absolute highest cross-fold validation accuracy, which therefore usually provides more features than “1se.”

To ensure model robustness and maximize the size of our training set, we used a modified five-fold cross-validation. We randomly shuffled and split all samples from all three cohorts into five equal-sized groups. For survival, we labeled “high risk” patients as those who suffered death/metastasis within 5 years, and “low risk” patients as those who had at least 5 years of uneventful follow-up. For metastasis, “high risk” patients were defined as those who suffered a metastasis at any time point, and “low risk” patients were metastasis-free for 5 years (for censored data) or beyond. Patients who were lost to follow-up (censored) before 5 years were not labeled as either high risk or low risk and removed for prognostic model training.

Traditionally in five-fold cross-validation, the feature weights (i.e., coefficients) generated by separate models are aggregated to provide a final model. This process is called “bagging.” Here, instead, we used four of five groups to train a model which was then used to predict risk labels on the fifth hold-out group as validation. By repeating the process five times, all patients were used to train and test models, but there was no overlapping of patient data during each training and testing. Patients were resampled so that there was an equal proportion of high and low risk labels, with the total number of labels for each class being equal to the larger class prior to resampling. The performance metrics (F1 score sensitivity and specificity) were calculated by concatenating the results for the test set of each fold into a single matrix. We implemented this cross-validation process for random forests (RF), support-vector machines (SVM), k-Nearest Neighbors (KNN), and logarithmic classification. In certain instances, two models yielded similar accuracy, but they do not have the same level of interpretability; for example, logarithmic classification is more interpretable than SVM and KNN. The coefficients from logistic regression for each CNN-derived dataset and LASSO-derived feature set are visualized to investigate whether image features receive consistent coefficients in multivariate survival stratification task (Supplementary Figures 4, 5). This is further discussed in the Results section.

### 2.5. Image feature visualization for interpretability

To visualize the features used for risk stratification, we generated “heatmaps” using the *ggplot2* (27) and *ggnewscale* (28). The heatmaps are placed side-by-side with the Hematoxylin and Eosin-stained WSI for inspection and direct interpretation by the pathologist.

### 2.6. Ethics statements

This study involves human subjects. The TCGA and CPTAC consortia provide their data to the public, and the data (follow up and histopathological images) is not linked to PHI. For the IUSM cohort of patients, secondary use of identifiable information and biospecimen is covered under our own broad institutional IRB.

## 3. Results

### 3.1. Assessment of GoogLeNet performance on tumor region identification

As described in the Methods section of this manuscript, four GoogLeNet CNNs were trained to recognize tumor tissue image patches. These four models were created using the CPTAC, TCGA, IUSM, as well as a balanced random selection of image patches from all three cohorts (“Merge”). All models perform well as indicated by the consistent high sensitivity and F1 scores across datasets (Figure 2D). For the few misclassified patches, visual inspection by our pathologist revealed that normal tissue predicted as tumor tended to contain mitotic figures, dense inflammation, and poor-quality image patches that remained despite our filtering process (Figures 2B, C).

### 3.2. Results of univariate analysis

#### 3.2.1. Univariate Kaplan-Meier survival analysis

**FIGURE 3:**
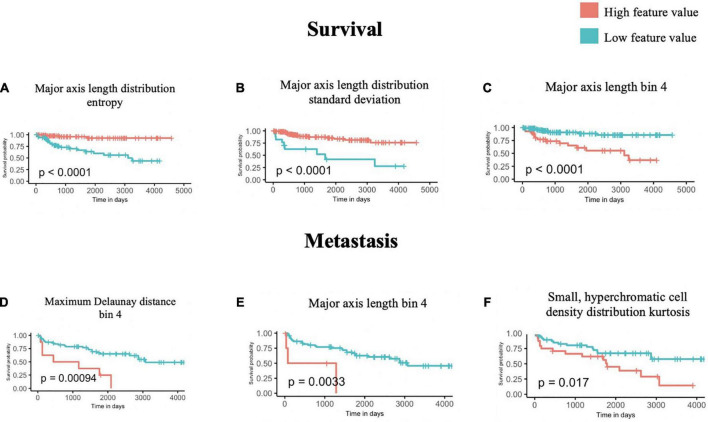
Univariate survival and metastasis analysis. After scanning through 100 cutoffs through the range in values for each feature, the cutoff providing the most significant *p*-values from log-rank tests of survival **(A–C)** and metastasis **(D–F)** times are used to generate Kaplan-Meier curves.

Using the univariate Kaplan-Meier log-rank test, we identified several features that can significantly stratify patients on their survival outcomes. The three most statistically significant ones are shown in Figures 3A–C, which are *Major axis length distribution entropy, Major axis length distribution standard deviation*, and *Major axis length bin 4*.

We further interpreted each of the identified features: As shown in Figure 3A, *Major axis length distribution entropy* significantly stratifies patient survival (log-rank *p* < 0.0001). Entropy is a measure of distribution uniformity, where high entropy represents a large variation in cell sizes, therefore, distributions with high entropy tend to have a high standard deviation. In Figure 3A, high entropy of the *Major axis length* distribution correlates with a better prognosis. This aligns with Figure 3B, which shows that a high standard deviation in the *Major axis length* also correlates with a good prognosis. Together, both features suggest that a high heterogeneity in cell sizes in a histopathological specimen (inflammatory, tumor, stromal, and otherwise) portend a better prognosis.

*Major axis length bin 4* appears as a significant feature for both survival and metastasis (Figures 3C–E), with the same direction of effect, where a high proportion of this feature contends poor prognosis. The interpretation of this feature is summarized in Section “3.4 Morphological features associated with 5-years survival/metastasis prediction.”

#### 3.2.2. Univariate Kaplan-Meier metastasis analysis

**FIGURE 4:**
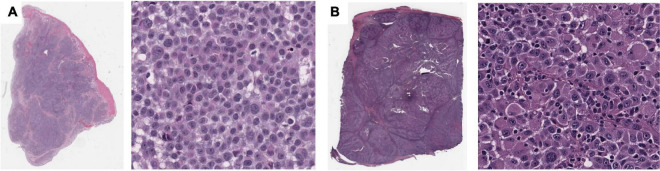
Feature visualization, *Maximum Delaunay distance bin 4*. At high power, **(A)** specimen TCGA-FR-A20 S, melanoma cells of dense/intermediate packing. **(B)** Specimen TCGA-ER-A19S, melanoma cells of very dense proliferation, going through stages of necrosis, and immune infiltration.

Using the same approach as above, we identified four features that are significantly associated with the prediction of 5-years metastasis (Figures 3D–F): *Maximum Delaunay distance bin 4, Major axis length bin 4*, and *Small-Hyperchromatic cell density distribution kurtosis*. *Maximum Delaunay distance bin 4* represents cells of intermediate packing density. High values of this feature were associated with a higher likelihood of metastasis in the univariate analysis. This feature is correlated with the *Minimum Delaunay distance bin 4* and *Mean Delaunay distance bin 4*, both of which are high risk for survival prediction: Spearman correlation coefficient (SCC) with *Maximum Delaunay distance bin 4* across all three datasets 0.55 and 0.899, respectively. We found that this density, defined by nuclei centroids, was seen in very different histomorphologies: In Figure 4, we show two regions with the similar density, but one is composed of small cells with intermediate packing Figure 4A, and the other is composed of distended rhabdoid cells (nuclei pushed to side of cell by cytoplasm) in a setting of very dense proliferation and immune infiltration Figure 4B.

**FIGURE 5:**
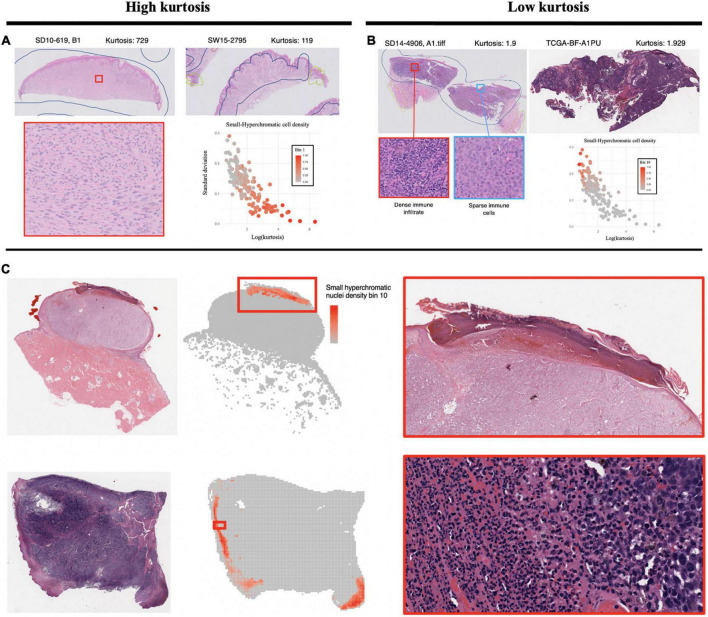
Visualization of small, hyperchromatic nuclei. **(A)** Demonstrates high kurtosis of Small-Hyperchromatic cells. These slides have uniformly low densities of immune infiltration. **(B)** Low kurtosis of Small-Hyperchromatic cells shows slides with a high variability of immune infiltration and necrosis across the entire specimen. Kurtosis of this feature is inversely correlated with standard deviation, and slides with high kurtosis have lower densities of Small-Hyperchromatic cells. In **(C)**, binl0 represents the highest density of Small-Hyperchromatic cells. Like the slides with low kurtosis in panel **(B)**, these high-density regions harbor necrosis of tissue with dense lymphocytic and neutrophilic infiltrate. Slides with low kurtosis have a higher standard deviation and higher density of Small-Hyperchromatic cells. The convolutional neural networks (CNN) used to filter out background patches in this visualization was trained on the clinical proteomic tumor analysis consortium (CPTAC) dataset.

**FIGURE 6:**
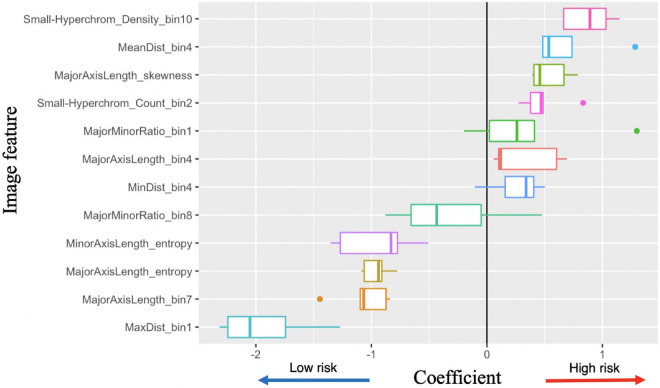
Image feature coefficients in 5-years survival logarithmic classifier based on Merge convolutional neural networks (CNN) and 1se LASSO feature set. Model weights for five logarithmic classification sub-models trained in the five-fold cross validation.

The *Small-Hyperchromatic cell distribution kurtosis* is a significant predictor in the Kaplan-Meier univariate analysis of metastasis (log-rank *P* = 0.017). Kurtosis measures the tailedness of a distribution. Further examination of slides with high kurtosis reveal specimen with a low density of inflammatory cell infiltration (mainly lymphocytes in this setting, Panel 5A). There is also a noted negative correlation between *Small-Hyperchromatic cell density kurtosis* and *standard deviation*, and we identify that slides with a high density of *Small-Hyperchromatic cells* tend to have distributions with low kurtosis and high standard deviation (Figures 5A, B). Aligning with kurtosis being a high-risk feature, *Small-Hyperchromatic cell distribution standard deviation* is a low-risk image feature in the multivariate survival models (Figure 6). Weak infiltration of tumors by lymphocytes is a well-established independent poor prognostic factor that pathologists assess (29), and for this reason, an image analysis pipeline for accurate quantification of tumor infiltrating lymphocytes has been studied (30).

To better understand what this *Small-Hyperchromatic cell* feature represents, we visualized the upper extreme of the density distribution (bin 10), demonstrating areas of necrosis and dense immune infiltration and ulceration on the peripheries of a nodular melanoma (Figure 5C). Ulceration occurs when tumors outgrow their blood supply and is an accepted marker for aggressive tumor biology and used for staging (31). As for the slides with low kurtosis (Figure 5B), they are associated with a high variability in the density of immune infiltration: In the same histologic specimen, there are regions with dense immune infiltration and necrosis, and other regions with sparse immune infiltration.

### 3.3. Multivariate risk stratification for 5-years survival and metastasis

As a baseline, stratifying patients based on their AJCC stage provided poor predictive values, with the F1 scores for survival and metastases being 0.44 and 0.51, respectively (Table 2). We experimented with several classification models using our image features, and several provided reasonable accuracy (Supplementary Table 1, Supplementary Figure 3). For 5-years survival prediction, the logistic classifier using the CNN trained on merged cohort for tumor region and Lasso-min feature set provided an F1 score of 0.72. For metastasis prediction, the KNN using the entire WSI and Lasso-1se feature set generated an F1 score of 0.73, while a comparable F1 score of 0.72 was achieved for the RF trained using the CNN trained on the IUSM cohort and Lasso-1se feature set (Table 3, Supplementary Table 1).

**TABLE 2:** Accuracy of American Joint Committee on Cancer (AJCC) to predict 5-years survival and metastasis, where stratification is by Stages I and II vs. Stage III.

Stages I and II vs. III	Sensitivity	Specificity	F1 score
Survival	0.455	0.735	0.444
Metastasis	0.414	0.778	0.511

**TABLE 3:** The best models for survival and metastasis prognosis, among convolutional neural networks (CNN)-trained tumor region-only and whole slide images (WSI), LASSO-derived feature sets, and classifiers.

Prognosis	Best model	Sensitivity	Specificity	F1 score
5-years survival	CNN on merged cohort, Lasso-min, logistic regression	0.86	0.78	0.72
5-years metastasis	CNN on IUSM cohort, Lasso-1se, random forest	0.78	0.57	0.72
	WSI, Lasso-1se, KNN	0.72	0.71	0.73

### 3.4. Morphological features associated with 5-years survival/metastasis prediction

#### 3.4.1. Image features associated with 5-years survival

With the successful predictions on 5-years survival and metastasis, we further examined the image features. Logistic regression has the best interpretability, because the coefficients learned for each image feature can be visualized (Figure 6). There are several features with very high or low risks. The highest risk morphology for the Merge CNN-derived logistic regression survival model using the 1se feature set was the *Small-Hyperchromatic cell density bin 10*, while the lowest risk phenotypes were the *Maximum Delaunay distance bin 1, Major axis length bin 7, and Major axis length distribution entropy*.

Because both WSI and CNN-derived tumor-region-only survival models achieved high accuracy, we visualized and examined the coefficients assigned to all image features among all survival models, with most features show consistent direction of effect for the decreased or increased risk hazard (Supplementary Figure 4). We also visualized coefficient weights stratified by tumor-region-only (CNN) vs. WSI-derived models to check whether any features were weighted oppositely if background stroma was included. We did not find this to be the case, and tumor-region-only and WSI coefficients were consistent in direction of hazard coefficients (Supplementary Figure 5).

#### 3.4.2. Image features associated with metastasis

The peak performance for metastasis is achieved by the KNN classifier using the entire the WSI and 1se LASSO feature set. This feature set contains: *Major axis length bin 4, Major axis length bin 7, Major minor ratio bin 1, Mean Delaunay distance bin 4, Max Delaunay distance bin 1, Small-Hyperchromatic cell count bin 2, Major axis length distribution skewness, and Major axis length distribution entropy.*

#### 3.4.3. Visualization and interpretation of identified image features

**FIGURE 7:**
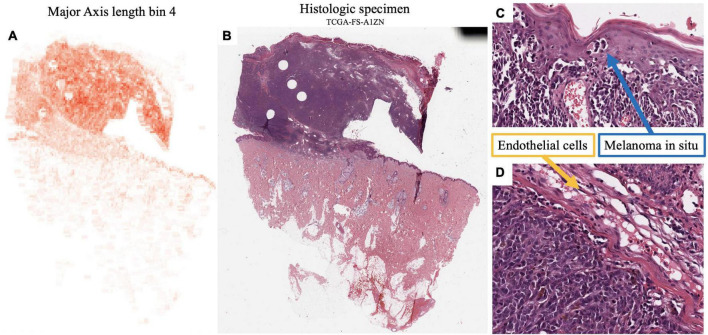
Feature visualization, Major Axis Length bin 4. **(A)** Major Axis bin 4 heatmap shown adjacent to the original **(B)** specimen. When inspected on high power, these cells are intermediate-to-small sized. **(C)** Melanoma in-situ with similarly sized spinosa cells. **(D)** Similarly sized endothelia. Tumor region identification by clinical proteomic tumor analysis consortium (CPTAC) convolutional neural networks (CNN).

*Major axis length bin 4*, mentioned previously, is a significant image feature for the univariate analysis of both survival and metastasis, with the same direction of effect: large values of this feature were associated with poor prognosis. This feature was maximized in specimen with small to intermediate sized melanocytes (Figure 7). Small cell melanoma has been associated with a poor prognosis previously in case series and case reports (32, 33), and our feature of *Major axis length bin 4* is consistent with this finding. Small cell melanoma, however is exceedingly rare, and though some IHC staining patterns of this variant have been described in patients with metastatic disease but of unknown primary lesions, it has not been systematically studied (34).

Additionally, *Major axis length bin 4* negatively correlated with *Major axis length standard deviation* (SCC-0.45). Not surprisingly, *Major axis length standard deviation* was also significant in the univariate analysis, where histologic specimen with low standard deviation was associated with poor prognosis, which also indicates the less variable melanocyte morphology on the H&E slides. *Major axis length distribution standard deviation* has a 0.892 SCC with *Major axis length distribution entropy*, which is a good prognostic feature used by the multivariate metastasis model. Therefore, the direction of effect of these features is consistent, and slides with many intermediate sized cells are associated with less variation in cell sizes across the entire specimen, which may indicate a poor prognosis for both survival and metastasis.

*Major axis length bin 7 is* correlated with a good prognosis in multivariate survival models (Figure 6). There is research to suggest that large nuclei are correlated with poor prognoses (35), which is slightly different from our features, because we segmented the entire cell rather than just the nucleus. As stated previously, small melanoma cells have also been associated with a poor prognosis. Enlargement of nuclei is typical in cancer histology. One hypothesis could be that *cell* enlargement to an extreme degree may represent a cell which replicates its DNA and cytoplasmic contents but cannot enter S phase and divide properly. Extremely large cells would therefore be a better prognostic factor. Macrophages, with their small nuclear to cytoplasmic ratio, could also contribute to this large-cell category. Our pipeline makes measuring small but systematic differences possible.

The *Small-Hyperchromatic cell distribution kurtosis* is also a significant predictor for univariate metastasis analysis and poor survival in multivariate survival analysis, which was already discussed in above univariate analysis section.

## 4. Discussion

The motivation behind this study is to develop an interpretable cell morphology pipeline and construct machine learning models for sensitive and specific 5-years survival (SN:86%, SP:78%) and metastasis (SN:72%, SP:71%) prognostics. We were able to generate several models that are highly sensitive and specific for both 5-years metastasis and survival risk prediction. Our work demonstrated that image features as the sole variables are powerful prognostic tools for prediction tasks, and the methodology is low cost, fast, and easy to implement.

We showed that the CNN-based approach used to isolate tumor regions improved predictive performance and reduced variability among classifiers in some instances. Moreover, no features extracted from only CNN-identified tumor regions had an opposite effect as the ones extracted from the whole slide images (Supplementary Figure 5). This demonstrated that the identified morphological descriptors are very robust to highly heterogenous cell quantities and morphologies in the histopathology slides. Given the enormous variety of melanoma histology and very small feature sets, we consider the sensitivity and specificity of this metastasis pipeline as promising for future development and adoption. Although the advantage of adopting the tumor selection step may not be obvious, we aim to test this same pipeline for our future cohort study: It will mostly contain patients with Stages I and II melanoma, and therefore, whose biopsies contain much more non-tumor tissue and very limited tumor region.

In this work, we discovered that the high density of *Small-Hyperchromatic cells* coincided with tumors that have more necrosis, ulceration, and pockets of dense inflammatory cell infiltration (Figure 5), and that cells with less immune infiltration overall, and the few that are present have a uniform distribution across the histological specimen. In specimen with greater degrees of immune infiltration, there is a large standard distribution of densities, characterized by pockets of dense inflammation and sparsely infiltrated areas. The density, kurtosis, and standard deviation of *Small-Hyperchromatic cell density* were all significant features for the prediction of metastasis and survival, and the direction of effect was consistent.

We found that slides with the densest regions of *Small-Hyperchromatic cells* coincide with large amounts of necrosis, especially ulceration, which is necrosis at the surface of the tumor (Figure 6C). Tumor ulceration is known as a poor prognostic factor for metastasis and survival, and what differentiates Stages IIa and IIb melanoma, and one of two criteria which differentiates Stages Ia/Ib. The interaction between the quantity and variability of immune infiltration and necrosis was not readily decipherable, and we hope to focus on this specifically in future research, by classifying cell types in the tumor and microenvironment, to quantify the colocalization of distinct inflammatory, stromal, and tumor cells directly.

Our model revealed that *Major Axis Length bin 4* was a significant feature used to predict both survival and metastasis. This feature corresponded to melanoma cells of intermediate to small size. Smaller melanoma cells have been reported to have a more aggressive clinical course (34). Larger cells (*Major Axis Length bin 7*) were associated with a better prognosis in our survival models. The relationship between cell size and prognosis in melanoma will be rigorously studied in a large cohort of patients in our future work.

Despite the successful development of machine learning models using interpretable features from primary biopsy histopathology for prognosis of melanoma, there are still limitations in our study: First, we had a limited cohort of Stage I/II patients, for whom a tool such as this would have the greatest impact. This is a problem common to melanoma metastasis research, more generally. Expanding our analysis to include clinicopathologic variables in a large cohort of Stage I/II is being carried out in our ongoing project. Additionally, though a good portion of the identified features for metastasis are interpretable or recapitulate those features known for survival prediction, for those that are still not discernable to human eyes, we believe that a large sample size will allow us to further validate and understand those features. For example, our pathologist was not able to identify a consistent pattern among cell morphologies among WSIs that maximized the *Minimum Delaunay distance skewness* feature, which may demonstrate that computer-quantified features are not always distinguishable to human eyes and may be superior to human in terms of refined feature extraction.

Third, the information captured by some features is correlated, and therefore may be redundant. For example, it is difficult to tell the difference between *Minimum Delaunay distance bin 10* and *Maximum Delaunay distance bin 1*. Rather than having three different distributions for maximum, minimum, and mean Delaunay distances among nuclei centroids, we can use a single distribution to describe cell density. Also, taken together, area and major/minor axis ratio together provide information about how large and ellipsoid a cell is, and the features *Major Axis* and *Minor Axis Length* may be redundant.

Finally, we did not explicitly model the interactions between specific cell types within the tissue. Existing research has quantified the architecture of the melanoma tumor and microenvironment by building “topological tumor graphs” that consist of a web of connected lymphocytes, fibroblasts, and cancer cells (36). Tumors with increased stroma and fibrous barriers separating lymphocytes from tumor were associated with a *worse* prognosis. Our work employs statistics (i.e., kurtosis, entropy, standard deviation) to describe the cell heterogeneity within a single histological specimen. We do not however, explicitly measure cell-cell interactions and spatial arrangements. Identifying cell types and establishing a metrics for their interactions is part of our ongoing work. In our future work, we plan to incorporate similar metrics into models to improve prognostic accuracy with a larger cohort.

This research has important implications for the future. Our research team has applied this interpretable cell morphology machine learning pipeline to several cancer types with success (17, 37). We have made improvements on the framework to improve model stability by reducing collinear variables and investigating the role of CNNs in focusing the analysis to tumor regions. The next step for our research is to assemble a large retrospective cohort of approximately Stages I and II patients with at least 5 years of clinical follow up. Being able to accurately predict 5-years metastasis risk in a large cohort of early-stage melanoma patients would transform melanoma clinical care. Currently, there is a shortage of dermatologists, and melanoma is a common, potentially aggressive cancer. This prognostic tool could help diagnose future melanoma metastasis at an earlier stage, which could potentially improve a patient’s chance of survival, as response to treatment in advanced melanoma is inversely correlated with tumor burden (38). Triaging early-stage patients would also provide researchers with a means to identify a patient population for studying the biology of metastasis and tumor dormancy.

Our pipeline could also be applied to the study of immunotherapy response. The current clinical gold standard is PD-L1 staining of tumor tissue, but it is poorly predictive of patients who will respond to immunotherapy, nor those who will have adverse events due to the immune checkpoint inhibition (39). AI approaches to predict these by analyzing histopathology and radiological images have been published, but most employ DL learning approaches and interpretability/explainability is still a key issue (40). As in our discussion of melanoma prognosis, we believe that deep-learning and more interpretable approaches are both needed for effective clinical translation.

## 5. Conclusion

In this study, we were able to develop two models, which use a set of interpretable morphological features, to predict melanoma 5-years survival and metastasis with maximum F1 scores of 0.72 and 0.73 respectively. The maximum sensitivity of our metastasis model is 0.72, and although this level of sensitivity is not superior to the published deep learning-based methods, our models are transparent on the features identified and are much more interpretable than deep-learning approaches. We demonstrated the interpretability of image features and models by recapitulating several known prognostic features. We believe that the accuracy of our metastasis model will improve with a larger cohort of patients. Overall, our methods proved quite interpretable and accurate, laying the foundation for a robust, clinically relevant, accurate, low-cost, and rapid metastasis prediction tool for early-stage melanoma that can complement deep-learning techniques.

## Data availability statement

The raw data supporting the conclusions of this article will be made available by the authors, without undue reservation.

## Ethics statement

The studies involving human participants were reviewed and approved by Indiana University School of Medicine. Written informed consent for participation was not required for this study in accordance with the national legislation and the institutional requirements.

## Author contributions

JC and ZL contributed equally to the work in this manuscript and analyzed the results and prepared the figures. JC performed the prognostic model training and wrote the manuscript. ZL trained the convolutional neural networks. AA and JC interpreted the histologic images. AA provided the IUSM cohort slides and follow-up information. JZ, AA, and KH formulated the questions and supervised the project. All authors contributed to the article and approved the submitted version.
