# Supplementary figures and images for: Predicting melanoma survival and metastasis with interpretable histopathological features and machine learning models
Source: Front Med (Lausanne). 2023 Jan 6;9:1029227. doi: 10.3389/fmed.2022.1029227 (PMC9853175; doi:10.3389/fmed.2022.1029227)

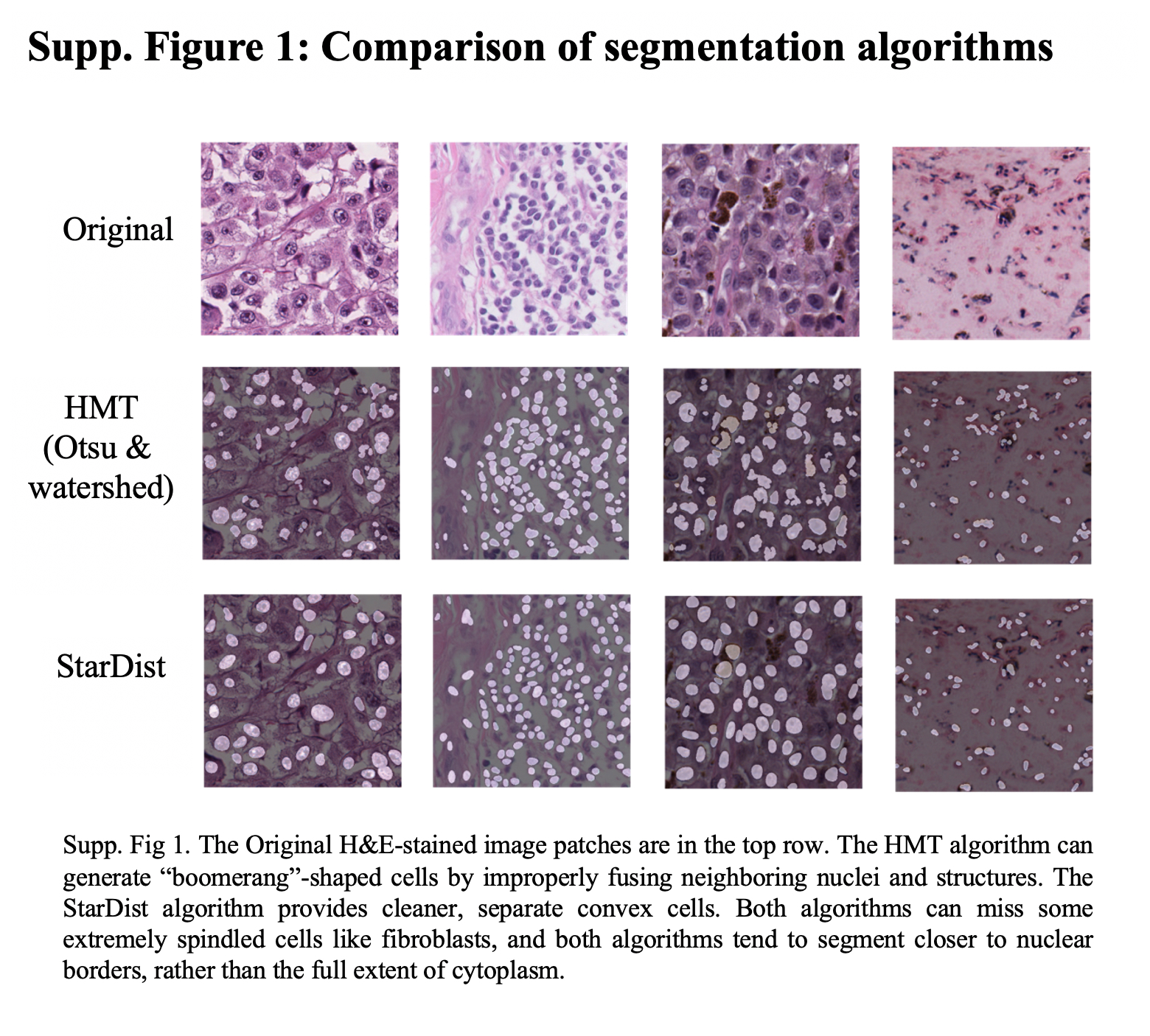

Supplement: Supplementary file 2 [file Image_1.jpeg]

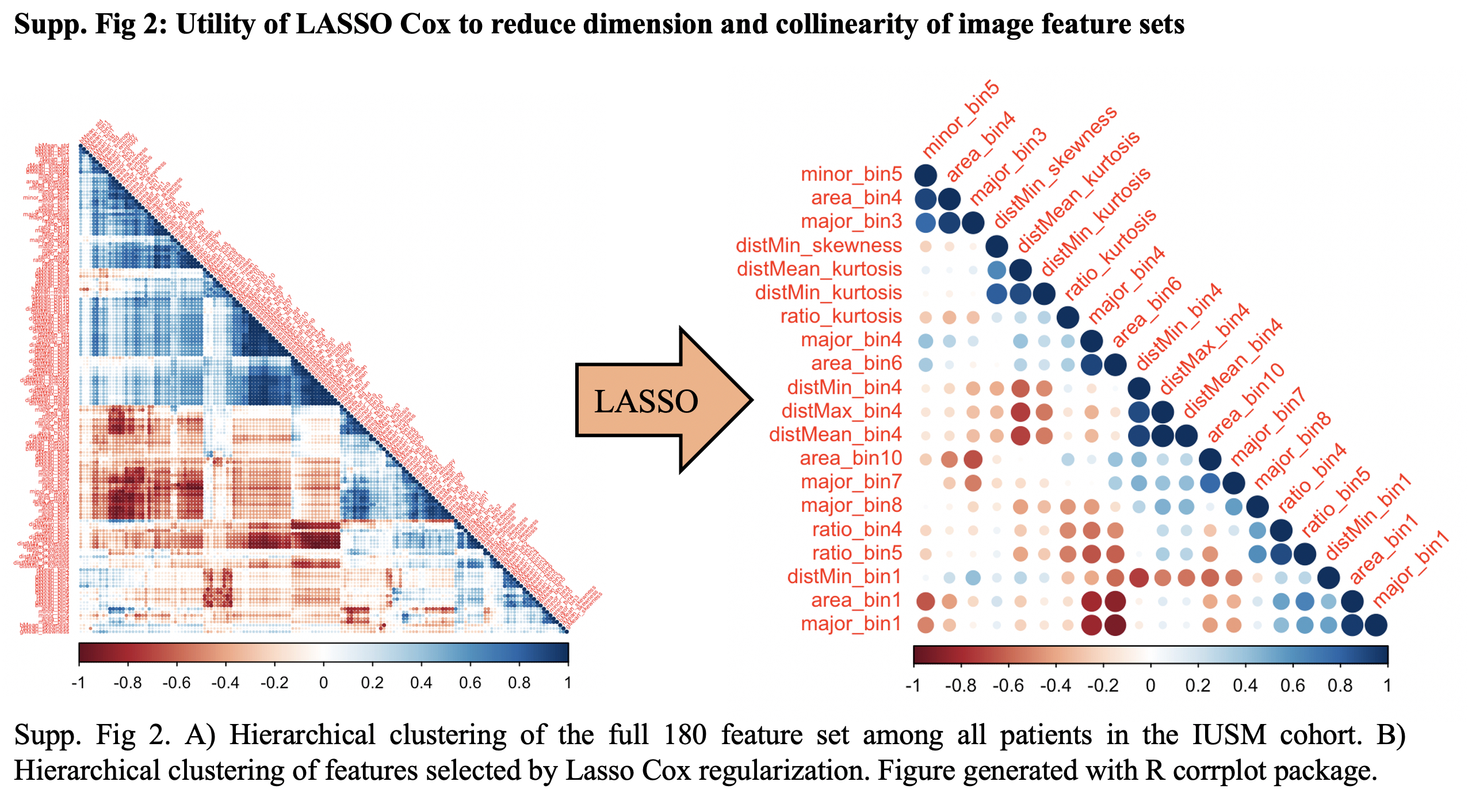

Supplement: Supplementary file 3 [file Image_2.jpeg]

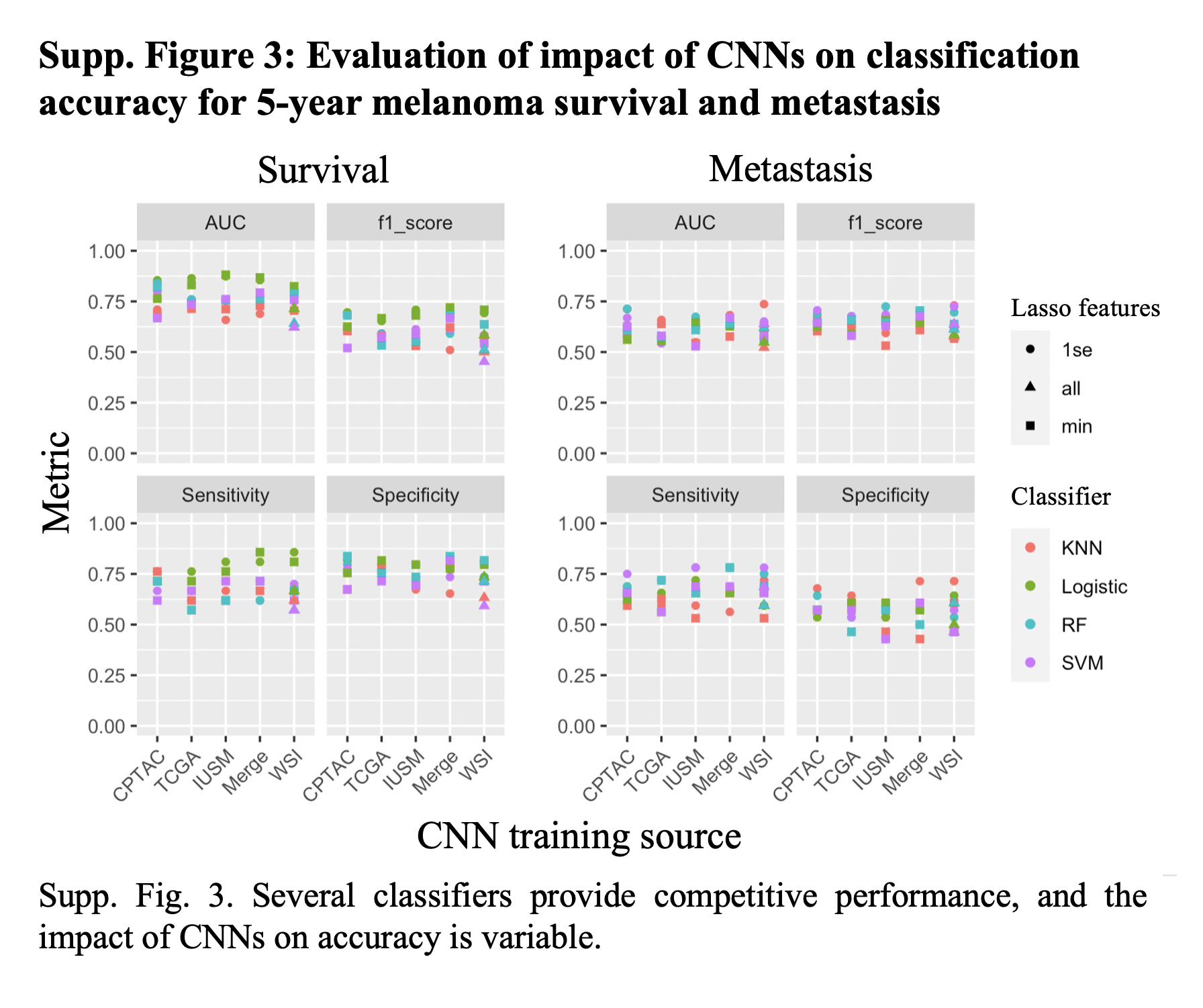

Supplement: Supplementary file 4 [file Image_3.jpeg]

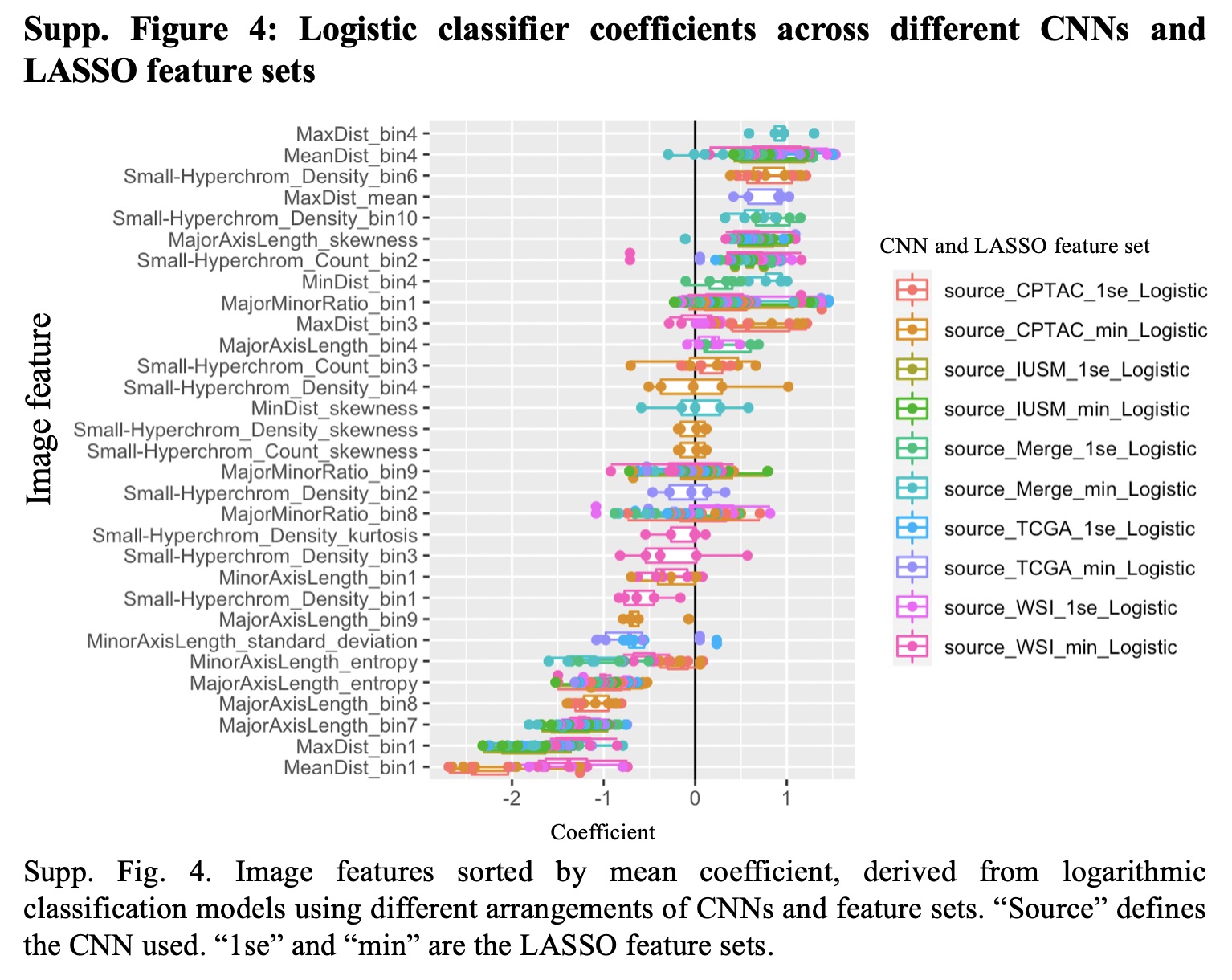

Supplement: Supplementary file 5 [file Image_4.jpg]

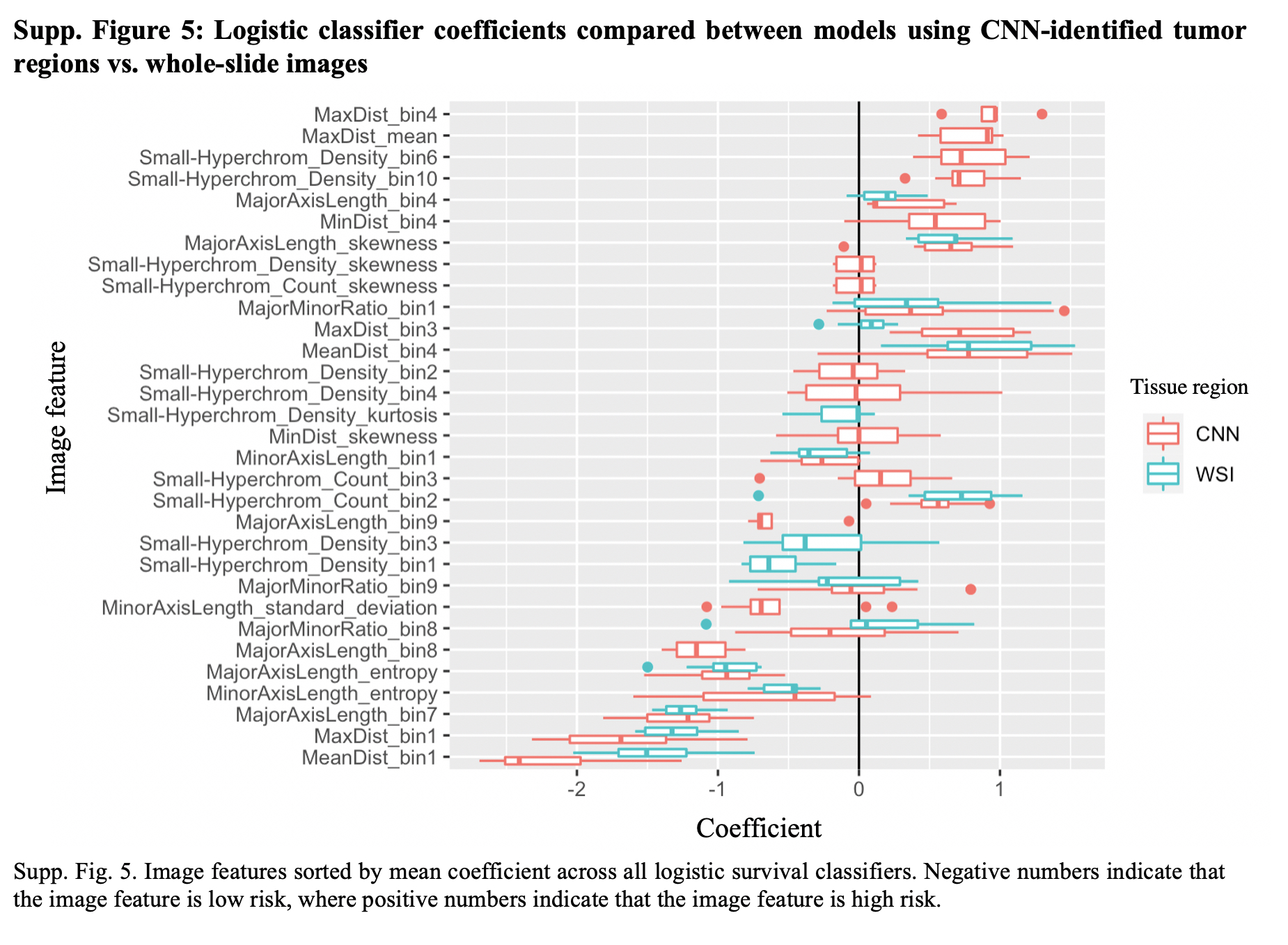

Supplement: Supplementary file 6 [file Image_5.jpeg]
